# The Role of Cluster of Differentiation 39 (CD39) and Purinergic Signaling Pathway in Viral Infections

**DOI:** 10.3390/pathogens12020279

**Published:** 2023-02-08

**Authors:** Alaa Elsaghir, Ehsan M. W. El-Sabaa, Abdulrahman K. Ahmed, Sayed F. Abdelwahab, Ibrahim M. Sayed, Mohamed A. El-Mokhtar

**Affiliations:** 1 Department of Microbiology & Immunology, Faculty of Pharmacy, Assiut University, Assiut 71515, Egypt; 2 Faculty of Medicine, Assiut University, Assiut 71515, Egypt; 3 Department of Pharmaceutics and Industrial Pharmacy, College of Pharmacy, Taif University, P.O. Box 11099, Taif 21944, Saudi Arabia; 4 Department of Biomedical and Nutritional Sciences, University of Massachusetts Lowell, Lowell, MA 01854, USA; 5 Department of Medical Microbiology and Immunology, Faculty of Medicine, Assiut University, Assiut 71515, Egypt

**Keywords:** ATP, CD39, CD73, CD39+ Tregs, COVID-19, HCV, HIV, influenza, viral infections

## Abstract

CD39 is a marker of immune cells such as lymphocytes and monocytes. The CD39/CD73 pathway hydrolyzes ATP into adenosine, which has a potent immunosuppressive effect. CD39 regulates the function of a variety of immunologic cells through the purinergic signaling pathways. CD39+ T cells have been implicated in viral infections, including Human Immunodeficiency Virus (HIV), Cytomegalovirus (CMV), viral hepatitis, and Corona Virus Disease 2019 (COVID-19) infections. The expression of CD39 is an indicator of lymphocyte exhaustion, which develops during chronicity. During RNA viral infections, the CD39 marker can profile the populations of CD4+ T lymphocytes into two populations, T-effector lymphocytes, and T-regulatory lymphocytes, where CD39 is predominantly expressed on the T-regulatory cells. The level of CD39 in T lymphocytes can predict the disease progression, antiviral immune responses, and the response to antiviral drugs. Besides, the percentage of CD39 and CD73 in B lymphocytes and monocytes can affect the status of viral infections. In this review, we investigate the impact of CD39 and CD39-expressing cells on viral infections and how the frequency and percentage of CD39+ immunologic cells determine disease prognosis.

## 1. Introduction

The cluster of differentiation 39 (CD39) is a 70–100 kDa transmembrane glycoprotein that is present in activated lymphocytes [1,2]. Kansas and colleagues characterized the expression and distribution of CD39, and they found that it is expressed on activated lymphocytes, endothelial and cancer cells, but not resting cells [3]. The level of CD39 and CD73 in T lymphocytes can predict the outcomes of certain cancers, such as chronic lymphocytic leukemia [4]. In addition, soluble CD39 was detected in human blood [5]. CD39 has ecto-(Ca^+2^-MG^+2^) apyrase activity that can hydrolyze ATP and ADP into AMP [6]. The binding of ATP to purinergic receptors (P2X and PY receptors) stimulates the purinergic signaling pathway, which is stimulated by infection and other inflammatory conditions [7]. ATP is degraded via CD39 into AMP that is further degraded into adenosine by the ecto-5′-nucleotidase enzyme of CD73, this pathway could affect the immune response against cancers [8,9,10,11,12]. Adenosine communicates through the G-coupled purinergic type 1 receptor (A1, A2A, A2B, and A3) and appears to antagonistically affect ATP binding sites [13,14]. Low concentrations of adenosine stimulate the chemotaxis of neutrophils via the action of A1 and A3 receptors, whereas high concentrations inhibit neutrophil trafficking and function via the action of A2A and A2B receptors [15]. Toll-like receptors (TLR) also upregulate the expression of adenosine 2b receptor on macrophages, which increases the interaction with adenosine leading to the downregulation of the immune response [16]. Following TLR activation, macrophages produce more ATP. This one is degraded by CD39 into adenosine, which inhibits macrophage inflammatory responses. Therefore, CD39 controls the autoregulatory mechanism of the macrophage function [17,18]. In addition, ATP and purinergic receptor signaling affect macrophage chemotaxis [19]. CD39 suppresses the chemotaxis of monocytes and macrophages and their functions [19]. CD39 plays a role in immunity against bacteria, parasites, and viruses. Additionally, it contributes to the progression of inflammatory diseases, allergic disorders, and cancers. In this review, we will focus mainly on the direct and indirect effects of CD39 on viral infections and how this marker affects the regulation of immune cells during viral infections.

## 2. CD39 Role in the Lymphocytic Subsets’ Functions during Viral Infections

CD39 significantly affects the function of different immune cells. CD8+ T lymphocytes are the most effective cells against viral infections, and it appears that their interaction with extracellular ATP is necessary for increased degranulation and cytotoxicity [20]. CD39 is a marker of T cell exhaustion that develops during chronic viral infection [21]. During chronic viral infections, CD39 and programmed cell death protein 1 (PD-1) present on CD8+ T lymphocytes are linked to the viral load [21]. CD39 and T cell immunoglobulin and mucin domain-containing protein-3 (TIM-3) define the exhausted CD8+ T lymphocytes in severe Corona Virus Disease 2019 (COVID-19) infection [22]. Similarly, CD39 determines the exhaustion status of CD8^+^ T cells and poor patient prognosis in cancers [23,24]. CD39 affects CD4+ T cell subsets such as T-regulatory cells (Tregs) and T-helper 17 (Th-17) cells. Tregs are differentiated from CD4+ T cells, distinguished by the presence of the forkhead box protein 3 (Foxp3) and CD25 [25,26]. Treg cells maintain immune homeostasis during viral infection by attenuating excessive antiviral and inflammatory responses and protecting vital organs from damage [27]. Tregs suppress the virus-specific T cell responses against some hepatotropic viruses, resulting in delayed viral clearance and induction of chronic liver inflammation [27,28]. CD39+ Tregs are a type of CD4+ Foxp3+ Treg associated with progressive viral infections such as HBV and HIV [29,30,31]. Dwyer et al. reported that the expression of CD39 on CD4+CD25+ T cells could differentiate between T-regulatory memory cells and other types of CD4+ lymphocytes, especially the ones that release interferon g (IFN-γ) and interleukin (IL)-17 cytokines [30]. Th-17 is a subset of the CD4+ T-helper cell lineage [32]. As discussed further in the review, Th-17 cells expressing CD39 are involved in particular viral infections, such as the influenza virus.

CD39 is also present in mouse regulatory γδ T cells [33]. CD39^+^ γδ T cells display CD25 but do not express PD-1, Foxp3, or CTLA-4, and they have an immunosuppressive effect through the production of IL-10 [33]. The frequency of CD39 on γδ T lymphocytes correlates with immune activation, CD4 count, and viral load. Moreover, CD39 and CD73 could identify the γδ T cells that secrete massive amounts of IL-10 [34].

CD39 is also essential for dendritic cell activity. These cells digest antigens in tissues and transport them to T cells-rich areas of secondary immune sites. CD39 is needed to efficiently activate hapten-reactive T cells on dendritic cells [35]. CD39 is believed to be essential for controlling the desensitization of P2 receptor sites required for dendritic cell activity by mechanically splitting ATP [35].

Additionally, CD39 is highly expressed on Natural Killer (NK) cells and Natural Killer T (NKT) cells [36]. CD39 is expressed on CD56+ NK cells and is involved in the pathogenesis of some viruses such as HIV [37]. CD39 regulates macrophage activity and chemotaxis [17,18].

The aforementioned data demonstrated that T cells and CD39 could both regulate the activity of innate immune cells.

## 3. CD39 Expression and RNA Virus Infections

Since CD39 is expressed on many immunologic cells, it is important in immune responses against RNA viruses (summarized in Table 1). The RNA viruses whose pathogenesis is affected by CD39 are listed below.

### 3.1. The Proportion and Role of CD39+ Cells during HIV Infection

HIV is a member of the genus *Lentivirus*, part of the *Retroviridae* family [56]. It is transmitted through sexual contact, blood transfusion, shared needle use during drug abuse, and vertical transmission from infected mothers to infants [57]. HIV infection’s primary targets are T-helper cells, where the vast majority of viral replication occurs. HIV causes human immune subversion and continuous loss of CD4+ T-helper cells, impairing the immune system and leading to acquired immunodeficiency syndrome (AIDS) [58].

Leal et al. reported that HIV infection enhanced ectonucleotidase activity and CD39 expression on lymphocytes, indicating that NTPDase and ATP hydrolysis are crucial to HIV infection [59]. CD39-positive lymphocytes are increased during HIV infection, resulting in continuous cellular activation and failure to develop memory cells [59]. However, specific anti-retroviral therapies did not alter this expression [60]. Interestingly, Barat et al. reported that HIV particles isolated from patients include CD39 in the envelope, affecting ATP metabolism and other physiological processes during HIV pathogenesis [61]. Another report revealed that the expression of CD39 on Foxp3+ Tregs was indicative of the disease status as CD39+ Treg cells correlated with the viral load, immune status, and disease pathogenesis [31]. Treg cells inhibited the replication of HIV in T cells through cAMP, which depends on the CD39/CD73 pathway [62]. A previous study reported that HIV-positive individuals have a higher ratio of CD39+ Tregs, and CD39/adenosine is vital in HIV pathogenesis. Blocking CD39 on Treg cells reduced the immune responses against HIV [63]. CD39+ Treg cells are inversely correlated with CD4+ T cell count in HIV-infected patients, suggesting that CD39 is crucial in AIDS progression [63]. According to the previous research findings, the expression of CD39 by Treg cells affects chronic HIV infection and disease progression, probably through its immunosuppressive metabolite (adenosine). The frequency of CD39+ Treg cells was increased during acute HIV infection, which was not affected by the initiation of anti-HIV therapy, and these cells could migrate to the gut and cause gut fibrosis [64]. CD39+ Treg cells suppress the activity of CD4+ T cells during HIV infection by inhibiting IL-2 [39]. Furthermore, CD39 can profile CD4 T cells into CD4+CD25+CD134+CD39+ Treg cells and CD4+CD25+CD134+CD39- T- cells, which determine the outcome of HIV infection and treatment responses [42]. Another study demonstrated that CD39 expression on naïve Treg (CD4^+^CD25^+^CD127^low^CD45RO^-^) correlated with HIV DNA in naïve HIV-infected patients, and these cells could be reservoirs for HIV during the chronic course [65]. In another subset of Treg cells, Fenoglio and colleagues reported that HIV infection upregulates the frequency of a certain CD8+ Treg subset: CD8^+^CD28^-^CD127^lo^CD39^+^ Treg cells [40]. The correlation between the frequency of these cells after anti-HIV therapy and immune status, HIV load, and clinical symptoms suggests that these cells could serve as a marker of response to anti-HIV therapy [40].

CD39 and PD-1 markers of exhausted CD8+ T lymphocytes are correlated with increased HIV load, chronic infection [38,66]. Interestingly, the suppression of CD39/adenosine and PD-1 signals can restore CD8+ T lymphocyte functions [38].

Regarding B cells, CD39+CD73+ B cells’ frequencies decreased in chronically naïve HIV-infected patients, correlated with HIV load, low CD4 count, and B cell activation [42].

During HIV infection, the expression of ectonucleotidase activity was upregulated in macrophages [67]. CD39 inhibition impairs HIV replication in human macrophages by increasing the level of extracellular ATP [67].

Besides, the expression of CD39 on NK cells affects HIV pathogenesis. In this regard, the frequencies of CD39^+^ CD56 ^bright^ NK cells, CD39^+^ CD56^neg^ NK cells, and CD38^+^CD39^+^ NK cells were correlated positively to HIV-viral load and negatively to CD4 count [37,68,69].

### 3.2. CD39/73 in Viral Hepatitis

#### 3.2.1. The Level of CD39+ Cells during Hepatitis C Virus (HCV) Infection

HCV belongs to the *Flaviviridae* family, and is surrounded by a core protein and an envelope containing two viral glycoproteins (E1 and E2) [70]. Patients with chronic HCV infection may develop cirrhosis, fibrosis, and liver cancer [71]. T cell responses are the primary host immune factors that determine the outcomes of HCV infection [72].

Tregs have been reported to be substantially increased in peripheral circulation and activated in individuals with HCV infections. Tregs had greater expression of CD39 and CD73, which are believed to be implicated in the development of HCV infection [44,73]. Although the level of Treg cells was higher in chronic HCV-infected patients, the percentage of CD39^+^ Treg cells was low in patients with advanced liver fibrosis [43]. Patients who cleared a primary HCV infection had an elevated level of CD4+ T cell effectors (CD25^high^CD134^+^CD39^-^) compared to Treg cells (CD25^high^CD134^+^CD39^+^) [44]. The percentage of Treg cells expressing CD39 and CD73 was decreased after achieving sustained virological responses with ribavirin and IFN-α [73]. Previous research findings indicate that CD39+ Treg cells contribute to determining HCV outcomes and disease prognosis. In addition to Treg cells, CD39 expression on HCV-specific CD8+ T cells is also documented. The expression of CD39 on terminally exhausted HCV-specific CD8+ T cells was correlated with the HCV load [21]. The level of CD39 on HCV-specific CD8+ T lymphocytes could differentiate between the cells directed against mutated antigens and cells directed against non-escaped mutants [45].

#### 3.2.2. The Level of CD39+ Cells during Hepatitis E Virus (HEV) Infection

HEV is a zoonotic pathogen that belongs to the *Hepeviridae* family [74]. HEV infection is transmitted through drinking contaminated water, contact with infected HEV reservoirs such as pigs or consumption of their products, transfusion of infected blood/blood products, and transmission from infected pregnant women to their offspring [75]. Most HEV infections are self-limited hepatitis. However, progression to acute liver failure is possible in pregnant women, old age, and patients with a history of liver disease [76]. Chronic HEV infection has been reported in patients with weakened immunity [77], and those patients could also develop other disorders beyond the liver subsequent to HEV chronicity [78]. Treg cells are involved in HEV pathogenesis where high T-reg cells (CD4^+^, Foxp3^+^, CD25 (^+/−^) were recorded in acute HEV-infected patients compared to those recovered and healthy subjects [79]. CD39 and CD73 have been identified in CD4+ Treg cells and are essential for the immunosuppressive activity of these cells [80]. Therefore, these markers are crucial for HEV infection. Treg cells exhibit high frequencies of CD39 and CD73 in acute HEV-infected patients compared to those recovered or healthy subjects [46]. Compared to healthy controls, Treg cells in recovered patients had a high frequency of CD73 but a low frequency of CD39 [46]. The expression of CD39 was negatively correlated with anti-HEV IgM in acute patients but not to alanine transaminase levels nor HEV RNA, indicating that CD39+ Treg cells are not associated with the complications of HEV infection [46]. CD39, but not CD73, was significantly increased in the stimulated peripheral blood mononuclear cells (PBMCs) versus non-stimulated cells isolated from acute HEV-infected patients [81]. The stimulation PBMCs collected from recovered patients and healthy controls with HEV recombinant ORF2 protein (rORF2p) was not associated with differences in the expression of CD39 or CD73 [81]. The previous findings have suggested that CD39+ Treg cells or CD39+ T-effector cells are crucial in HEV infection outcomes.

#### 3.2.3. CD39+ Cells and Hepatitis D Virus (HDV) Infection

HDV is a small single-stranded RNA-enveloped virus. It is a satellite virus that belongs to the genus *Deltaviridae* that requires HBV for infection, replication, and assembly of new virions [82]. HDV infection is either a coinfection with HBV or a superinfection on HBV-infected patients leading to fulminant hepatitis, chronic liver disease, cirrhosis, fibrosis, or hepatocellular carcinoma [83,84]. The fact that HDV increases the risk of HBV infection contributed to its discovery [85]. Extracellular ATP binds to P2X receptors and induces immune responses to inhibit infection [86,87]. P2XR activity is required for HDV infection of primary human hepatocytes [47]. P2X7 is also involved in the inflammatory responses (hepatitis) associated with HBV and HDV infection in the liver [47].

### 3.3. CD39+ Cells and Influenza Infection

Influenza A is a segmented RNA virus belonging to the family *Orthomyxoviridae* [88]. Influenza virus infection causes epidemics, outbreaks, and pandemics, as the virus is a highly infectious pathogen leading to global morbidity and mortality annually [89]. Influenza A virus includes two glycoproteins, hemagglutinin (HA), and neuraminidase (NA), which are responsible for viral entry and dissemination, and the reassortment between these two proteins leads to outbreaks [88,90]. Although the respiratory epithelium includes the two ectonucleotidases (CD39 and CD73), they are not required for acute lung injury [91,92]. The accumulation of ATP and its catabolic metabolite “adenosine” in mice lungs was not related to CD73 but to the alkaline phosphatase level [91,93]. However, Leyva-Grado and colleagues reported that the purinergic receptor P2X7 mediates the lung immunopathology of influenza virus infection [94].

Experienced memory Treg cells could control influenza virus infection in the lung, and these cells express a high level of CD39. CD39 and other markers such as CD69, CD103, and CTLA-4 discriminate experienced memory Treg cells from the inexperienced naïve Treg cells [48]. Hall et al. reported the treatment of influenza-infected females with progesterone-induced lung repair via several mechanisms, including increasing the number of regulatory Th17-expressing CD39 cells, suggesting a protective effect of CD39 against lung damage [49]. It was found that influenza vaccination among HIV-infected pregnant women resulted in heterogenic T cell responses, including CD4^+^CD39^+^ and CD8^+^CD39^+^ Treg cells [95,96].

### 3.4. CD39 and Dengue Virus Infection

Dengue virus is an RNA virus that belongs to the *Flaviviridae* family [97]. It is an arboviral infection transmitted by biting infected mosquitoes of the Aedes species [98]. Dengue virus infection can cause epidemics, resulting in dengue hemorrhagic fever with an elevated mortality rate [99]. The purinergic signaling pathway plays a role during dengue virus infection [100]. Extracellular ATP was found to decrease the viral load in vitro, and P2X7R signaling increased antiviral responses by increasing nitric oxide or inflammatory cytokines in human monocytes [100]. Likewise, ATP and P2X7R controlled the host immunity mediated by γδ T cells against Dengue virus-infected dendritic cells through regulation of IFN-γ [50]. Additionally, dengue virus infection reduced the expression of CD73 in endothelial cells, and recovery of endothelial barrier homeostasis was modulated by the expression of CD73 [101].

### 3.5. CD39+ Cells and Zika Virus Infection

Zika virus (ZIKV) is a member of the *Flaviviridae* family that is transmitted via mosquito bites, sexual intercourse, blood transfusion, and from pregnant women to their unborn babies [102]. ZIKV-infected patients develop no or mild symptoms, including fever, muscle, and joint pain, conjunctivitis, and skin rash. ZIKV causes abnormalities in the brain of infants, most frequently microcephaly [102]. During acute ZIKV infection, the level of Treg cells expressing CD39/CD73 was significantly higher in the infected patients [51]. CD39/CD73 Treg cells induce the hydrolysis of ATP/ADP into adenosine which mediates anti-inflammatory responses [51].

### 3.6. CD39+ Cells and Lymphocytic Choriomeningitis Virus (LCMV) Infection

LCMV is a segmented RNA virus that belongs to the *Arenaviridae* family [103,104]. The virus encodes a structural protein, and a nucleoprotein (NP) which is required for viral RNA polymerase and the formation of viral capsid [103]. The L-protein of LCMV contributes to its replication as a component of arenavirus polymerase [103]. Mice and rodents are the main reservoirs of LCMV, and infection can be transmitted to humans via contact with the rodents or their bites. Moreover, vertical transmission of LCMV has been documented to be associated with congenital disorders [105]. LCMV causes neurologic disorders in children and adults [105]. The expression of CD39 in the CD8+ T lymphocytes differed in acute and chronic LCMV infections [21]. CD39 and PD-1 were not expressed on naïve CD8+ T lymphocytes, and they started to be expressed following LCMV infection [21]. CD39 is a sign of CD8 terminally exhausted cells and contributes to chronic LCMV infection [21]. In the exhausted CD8 cells, CD39 is expressed with another inhibitory molecule, PD-1 [21]. Memory T lymphocytes from the central nervous system (CNS) of mice infected with LCMV expressed CD39, which correlated with T lymphocyte exhaustion [106]. The expression of CD39 in these cells did not change with time, and its expression in these cells correlated with active infection [106]. ATP-adenosine axis through CD39 can regulate the outcome of T lymphocytes and is associated with long-lived memory T cells [106].

### 3.7. CD39+ Cells and Severe Acute Respiratory Syndrome (SARS) CoV-2 Infection

Coronavirus is a positive-sense, single-stranded, enveloped RNA virus that belongs to the *Coronaviridae* family [107,108,109]. It causes acute respiratory syndrome (SARS-CoV-2) manifested by fever, general weakness, gut disturbances, cough, pneumonia, hypoxemia, and pulmonary edema [110,111,112]. SARS-CoV-2 spike proteins attach to cellular membrane receptors such as angiotensin-converting enzyme 2 (ACE2) [113,114] and CD147 [115,116]. In COVID-19 patients, there are changes in the purinergic signaling pathways and inflammatory cytokine levels. The level of CD39 and CD73 was elevated in total leukocytes with higher ATP and cytokine levels [117]. During COVID-19 progression, the alteration of CD39/CD73 was reported and correlated with disease severity [54]. The level of cytokines such as IL-6, IL-10, and IL-17a was higher in severe COVID-19 compared to mild disease and healthy controls [54]. However, the levels of ATP and adenosine were in the opposite direction [54]. The frequency of CD4+CD25−CD39+ (memory T-effector cell) was higher in severe cases than in mild cases and healthy controls, while the percentage of CD4+CD25−CD73+ T cells was comparable in all groups [54]. In contrast, the percentage of CD4+CD25+CD39+ (activated/memory Treg) was lower in infected cases and a much lower level in severe cases compared to mild ones [54].

The percentage of CD39+ cells in CD4+ and CD8+ T lymphocytes was higher in severe SARS-CoV2-infected patients than in mild patients and healthy individuals, but the percentage of CD39+ in monocytes (CD39+ CD14+) among these groups was comparable [52]. Nevertheless, this trend was not detected in CD73+ T cells among these groups [52]. The gene expression of several ectonucleotidase transcripts associated with CD39 (ENPP1, ENPP2, and ENPP3) was lower in severely infected patients than in controls [52]. The frequency of CD39+ in B lymphocytes was reduced in severe COVID-19 infection [52]. Another study revealed that the plasma concentration of soluble CD39 was elevated in COVID-19 patients and was associated with the length of hospital stay [55]. In addition, COVID-19 patients had elevated levels of CD39 in CD4+ and CD8+ lymphocytes, NK cells, Tregs, and monocytes, but not CD19+ B cells [55]. The higher expression of CD39 was associated with hypoxemia and innate immune responses [55]. These results revealed abnormalities in purine metabolism and altered CD39 expression in immune cells during COVID-19 disease.

**Figure 1:**
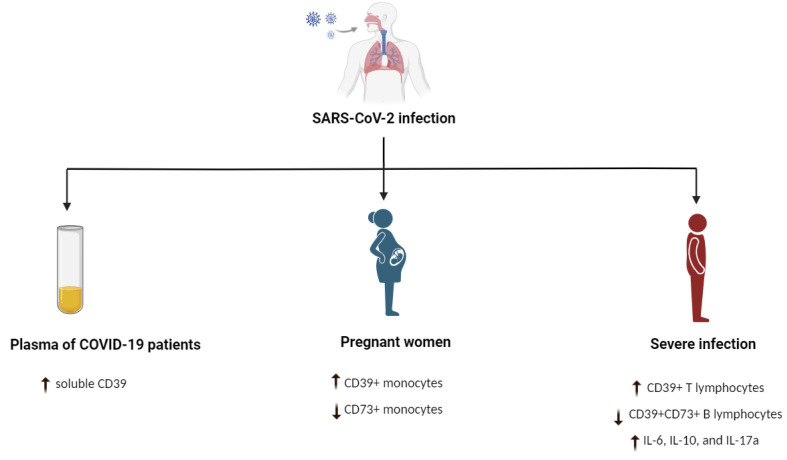
The link between CD39 and SARS-CoV2 pathogenesis. ↓ means decrease, reduction or suppress, ↑ means increase or activate.

During pregnancy/COVID-19 coincidence, the frequency of CD39 or CD73 in T lymphocytes or B lymphocytes was not changed. However, pregnant women (COVID-19 positive) had a greater proportion of CD39+ monocytes than non-pregnant women COVID-19 positive) [53]. Conversely, the CD73+ monocyte percentage decreased in COVID-19-positive pregnant women [53]. The difference in the frequency of these cells was associated with a difference in the inflammatory responses [53]. Another study showed that the expression of CD39, but not CD73, was downregulated in monocytes of infected patients compared to healthy subjects [118]. These results indicate that CD39-positive cells are involved in COVID-19 infection (Figure 1).

## 4. CD39 + Cells and Infections Caused by DNA Viruses

CD39+ lymphocytes also contribute to the pathogenesis of some DNA viruses (summarized in Table 2).

### 4.1. CD39+ Cells and Cytomegalovirus (CMV) Infection

CMV is a member of the *Herpesviridae* family [127]. CMV infection is transmitted via contact with fluids and mucous membranes, blood transfusion, sexual contact, and vertical to the offspring [127]. CMV infection has upregulated ecto-CD39, and CD73 endothelial cells to regulate platelets’ functions [119]. These ectoenzymes also control the superoxide secretion by the immune cells, thereby controlling inflammation [119]. High CD39 is also expressed on Treg cells (CD25^+^ high) that suppress the CD4+ T-effector function, and these cells mediated CMV recurrence or lasting infection, especially in kidney transplant patients [120]. CD8+ T cells specific to CMV infection do not exhibit CD39 as an indicator of exhaustion [21,128].

### 4.2. CD39+ Cells and Epstein–Barr Virus (EBV) Infection

EBV belongs to the *Herpesviridae* family [129] and causes latent and productive infection in B-lymphocytes and oral epithelium [130]. EBV infection can be associated with cancers such as Hodgkin and non-Hodgkin lymphomas, Burkitt lymphoma, and nasopharyngeal carcinoma [131]. During EBV infection, CD39 plays a role in lymphocyte signaling and adhesion. It is expressed on infected cancer B cells but not on non-infected B cells [3]. HIV infection increases EBV-associated B- cell lymphoma by reducing the B cell activation markers such as CD39 and CD23 [121]. CD39 is a marker of Burkitt’s lymphoma cells that develop lymphoblastoid phenotype [122]. Lymphoblastoid Cell Lines (LCL), which are developed by the transformation of B-cells by EBV, express a high level of CD39 and ATPase activity [132]. CD39 causes loss in the purinergic receptor (P2X7 nucleotide receptor) in the LCL due to the ATPase activity of CD39 [132]. EBV infection increased the number of CD39^+^ T-reg cells [123]. CD8+ specific T cells directed against EBV infection present in the tumor are heterogeneous. They did not express PD-1 but consistently expressed CD39, suggesting that exhausted T lymphocytes are present in EBV-driven cancer [133].

### 4.3. CD39+ Cells and Human Papillomavirus (HPV)

HPV includes a circular genome that belongs to the *Papillomaviridae* family [134]. The virus causes warts in the cutaneous and mucosal epithelium and can progress to cancers such as cervical cancer [135]. HPV16 and HPV18 strains cause most cervical cancers, and the transmission is caused by sexual intercourse [136]. Mice infected with HPV develop cervical cancer via B-cells, which promote cancer growth. The B cells in the tumor area exhibited decreased MHC and CD86 expression and increased CD39 and PD-L1 expression [124]. Cervical cancer cells infected with HPV expressed CD39 and CD73, which aided in tumor survival via escaping from the immune cells [125]. In a parallel line, a recent study reported that HPV16 increased the level of CD39 and CD73 in cervical intraepithelial neoplasms via TGF-β, which mediated immunosuppression and cancer development [137]. Patients with HPV16-associated oropharyngeal squamous cell carcinoma who express CD161 on their CD4 effector T cells have a higher survival rate [138]. Notably, the expression of CD161 was inconsistent with the expression of CD39 and PD1 [138].

### 4.4. CD39+ Cells and Hepatitis B Virus (HBV) Infection

HBV is a member of the *Hepadnaviridae* family [139]. It causes acute and chronic infections that could lead to liver cirrhosis, fibrosis, and cancer [140]. CD39+ expression on Foxp3+CD4+ T cells was associated with the progression of HBV [29]. The percentage of CD39+ Tregs was lower in chronically infected and liver-failure patients compared to healthy individuals [29]. Levels of CD39 and CD73 on B lymphocytes were inversely related to viral load and liver inflammation in chronic HBV-infected patients [126].

## 5. CD39-Positive Cells and HIV/Tuberculosis (TB) Coinfection

CD39 can predict mortality and pathogenesis of HIV/TB coinfection. Compared to HIV/TB coinfected patients who survived, the level of CD39 in CD8+ T lymphocytes was significantly higher in patients who died [141]. During HIV/TB coinfection, there are two categories of phenotypically Treg cells characterized: unconventional Treg (uTreg) (CD4+CD25-FoxP3+) and conventional Treg (cTreg) (CD4+CD25+Foxp3+) [142]. Interestingly, cTreg had significantly higher CD39 expression than uTreg in HIV/TB and healthy subjects but not in HIV monoinfection [142]. Additionally, uTreg cells produced IFN-γ more than cTreg in HIV/TB coinfected patients [142].

## 6. Conclusions and Future Perspectives

The CD39/CD73 axis and associated purinergic signaling pathways are crucial during viral infections. The effect of the CD39/CD73 axis may vary depending on the immune cells that interact with each virus and express CD39 for each infection. For example, CD8+ CD39+ T cells define the exhaustion status of HIV and HCV infections but not CMV or EBV infections. Another example is the expression of CD39 on B lymphocytes, which is not always related to the pathogenesis of viral infections. However, several details are lacking. Other viral infections, such as hepatotropic viruses, Ebola, Zika, and Dengue, have not been studied as extensively as HIV and COVID-19. Consequently, the impact of CD39+ positive immune cells on these viral infections is not fully understood. Additionally, endothelial cells express CD39. However, the effect of these cells on viral infections has not yet been investigated. Besides, CD39 can profile CD4+ T lymphocytes into effector cells and regulatory cells, which can determine the disease status. The role of effector T cells and T regs on some viral infections requires further research.

## Figures and Tables

**Table 1:** The expression of CD39 and purinergic receptor signaling in infections caused by RNA viruses.

Virus	Family	Virology	Main Findings Related to CD39+ Cells	References
HIV	*Retroviridae*	Two copies RNA, +ve sense Enveloped	CD8+ T exhaustion and chronic infection.	[21,38]
			↓ the activity of CD4+ T cells	[39]
			Marker for response to anti-HIV therapy	[40]
			CD39⁺ T reg cells are important for disease status and therapy.	[41]
			CD73+ B memory cells ↓ in chronic infection	[42]
HCV	*Flaviviridae*	Single-stranded RNA, +ve sense Enveloped	Associated with chronic HCV infection	[43,44]
			CD8+ T cell exhaustion	[21]
			Profile CD4 T cells into T-effector cells and Treg cells	[44]
			CD39^hi^ is associated with terminally exhausted CD8+ T cells. CD39^low^ is associated with memory T- cell	[45]
HEV	*Hepeviridae*	Single-stranded RNA, +ve sense Enveloped	CD39+ Treg lymphocytes ↑ in acute infection	[46]
			CD39+ T-effector lymphocytes were higher in acute infection than in recovery	[46]
HDV	The *Deltaviridae* genus does not belong to a known family	Single-stranded RNA, +ve sense Enveloped	Suramin and brilliant blue G (BBG) inhibit HDV and HBV infections via blocking of purinergic receptors	[47]
Influenza	*Orthomyxoviridae*	Segmented RNA, −ve sense Enveloped	↑ expression of CD39 on memory T reg cells and Th17 → control the infection and lung damage	[48,49]
Dengue virus	*Flaviviridae*	Single-stranded RNA, +ve sense Enveloped	Treating the infected dendritic cells with P2X7 receptor antagonist → ↓ IFN-γ response of γδ T against the virus	[50]
Zika virus	*Flaviviridae*	Single-stranded RNA, +ve sense Enveloped	CD39+CD73+ Treg cells ↑ in acute infection	[51]
Severe Acute Respiratory Syndrome Corona Virus-2 (SARS-CoV-2)	*Coronaviridae*	Positive sense, single-stranded RNA Enveloped	CD39+ T lymphocytes ↑ in a severe infection CD39+CD73+ B lymphocytes ↓ in severe infection	[52]
			Higher CD39+ monocytes in pregnant women infected with COVID-19	[53]
			↑ CD39+ memory T-effector cells and ↓ CD39+ Treg cells in COVID-19 cases	[54]
			↑ plasma CD39 in COVID-19 patients ↑ expression on effector T cells, Treg cells, NK cells, and monocytes Linked to hypoxemia and innate immune response. ↑ extracellular ATP, ADP → inflammation Ticagrelor (P2Y12 receptor blocker) ↓ COVID-19 plasma associated platelet activation. Overexpression of CD39 → thromboinflammation	[55]

↓ means decrease, reduction or suppress, ↑ means increase or activate, and → means lead to, correlate with, or linked to. −ve sense: negative sense, +ve sense: positive sense.

**Table 2:** CD39 expression and purinergic receptor signaling and infections caused by DNA viruses.

Virus	Family	Virology	Main Findings Related to CD39+ Cells	References
CMV	*Herpesviridae*	Double-stranded DNA Enveloped icosahedral capsid	↑ expression of CD39 on endothelial cells → regulates the platelet function and inflammation	[119]
			↑ expression on CD25+ Treg cells → recurrence and/or latent infection	[120]
EBV	*Herpesviridae*	Double-stranded DNA Enveloped icosahedral capsid	Expressed on EBV-infected cancer B cells, Burkitt lymphoma cells, and LCL	[3,121,122]
			EBV ↑ CD39+ Treg cells	[123]
HPV	*Papillomaviridae*	Double-stranded DNA Non-enveloped icosahedral capsid	↑ expression of CD39 on the B cells inside the tumor	[124]
			↑ expression of CD39 and CD73 on cervical cancer cells infected with HPV → immune escape	[125]
HBV	*Hepadnaviridae*	Double-stranded DNA Enveloped icosahedral capsid	A lower percentage of CD39+ Treg cells in HBV-infected patients than in healthy controls	[29]
			The expression CD39/CD73 in B cells is negatively associated with liver inflammation	[126]

↑ means increase or activate, and → means lead to, correlate with, or linked to.

## Data Availability

Not applicable.

## Abbreviations

ACE2: angiotensin-converting enzyme 2; AIDS: acquired immunodeficiency syndrome; ADP: adenosine diphosphate; AMP: adenosine monophosphate; ATP: adenosine triphosphate; PBMCs: peripheral blood mononuclear cells; CD: cluster of differentiation; CMV: cytomegalovirus; CNS: central nervous system; COVID-19: Corona Virus Disease 2019; CTLA-4: cytotoxic T-lymphocyte-associated protein 4; EBV: Epstein–Barr virus; ENPP: ectonucleotide pyrophosphatase/phosphodiesterase; ENTPD1: ectonucleoside triphosphate diphosphohydrolase 1; HA: hemagglutinin; FOXP3: forkhead box protein 3; HBV: Hepatitis B virus; HCV: Hepatitis C virus; HPV: human papillomavirus; IFN: interferon; IL: interleukin; LAG-3: lymphocyte-activation gene 3; LCMV: lymphocytic choriomeningitis virus; LCLs: lymphoblastoid B-cell lines NA: neuraminidase, NK cells: natural killer cells; NKT cells: natural killer T cells; PD-1: programmed cell death protein 1; TB: tuberculosis; TGF-β: transforming growth factor-beta; TLR: toll-like receptor; TIM-3: T cell immunoglobulin and mucin domain-containing protein-3; Th17 cells: T-helper 17 cells; Treg: T-regulatory cells; cTreg: conventional Treg; uTreg: unconventional Treg; ZIKV: Zika virus.
